# Balanced Synaptic Input Shapes the Correlation between Neural Spike Trains

**DOI:** 10.1371/journal.pcbi.1002305

**Published:** 2011-12-22

**Authors:** Ashok Litwin-Kumar, Anne-Marie M. Oswald, Nathaniel N. Urban, Brent Doiron

**Affiliations:** 1 Program for Neural Computation, Carnegie Mellon University and University of Pittsburgh, Pittsburgh, Pennsylvania, United States of America; 2 Center for the Neural Basis of Cognition, Pittsburgh, Pennsylvania, United States of America; 3 Department of Biology, Carnegie Mellon University, Pittsburgh, Pennsylvania, United States of America; 4 Department of Mathematics, University of Pittsburgh, Pittsburgh, Pennsylvania, United States of America; Indiana University, United States of America

## Abstract

Stimulus properties, attention, and behavioral context influence correlations between the spike times produced by a pair of neurons. However, the biophysical mechanisms that modulate these correlations are poorly understood. With a combined theoretical and experimental approach, we show that the rate of balanced excitatory and inhibitory synaptic input modulates the magnitude and timescale of pairwise spike train correlation. High rate synaptic inputs promote spike time synchrony rather than long timescale spike rate correlations, while low rate synaptic inputs produce opposite results. This correlation shaping is due to a combination of enhanced high frequency input transfer and reduced firing rate gain in the high input rate state compared to the low state. Our study extends neural modulation from single neuron responses to population activity, a necessary step in understanding how the dynamics and processing of neural activity change across distinct brain states.

## Author Summary

Neurons in sensory, motor, and cognitive regions of the nervous system integrate synaptic input and output trains of action potentials (spikes). A critical feature of neural computation is the ability for neurons to modulate their spike train response to a given input, allowing task context or past history to affect the flow of information in the brain. The mechanisms that modulate the input-output transfer of single neurons have received significant attention. However, neural computation involves the coordinated activity of populations of neurons, and the mechanisms that modulate the correlation between spike trains from pairs of neurons are relatively unexplored. We show that the level of excitatory and inhibitory input that a neuron receives modulates not only the sensitivity of a single neuron's response to input, but also the magnitude and timescale of correlated spiking activity of pairs of neurons receiving a common synaptic drive. Thus, while modulatory synaptic activity has been traditionally studied from a single neuron perspective, it can also shape the coordinated activity of a population of neurons.

## Introduction

Correlations between the spike trains of neuron pairs are observed throughout the central nervous system [1]. The correlation between a pair of neurons' spike trains can change depending on the state of their neural circuit. For instance, correlated neural activity is altered by stimulus properties [2], [3], anesthetics [4], [5], stimulus adaptation [6], focus of spatial attention [7], [8], and the behavioral context of a task [9]. The level of spike train correlation between neuron pairs has implications for the accuracy of population codes [10], the formation of neural assemblies [11], and the propagation of neural activity [12], [13]. Nonetheless, only recently has attention been given to the mechanisms by which correlated activity is modulated [14], [15], [16], .

Cortical neurons receive a mixture of excitatory and inhibitory synaptic inputs, resulting in spiking activity that is driven by input uctuations rather than the input mean [21], [22]. This state is often described as *balanced*, to denote that the mean excitatory and inhibitory inputs that neurons receive are approximately equal [23], [24]. Balanced activity is inuenced by stimulus properties and history [25], [21], as well as internal brain state [26]. These changes can modulate the integration properties of single neurons, strongly inuencing neuronal activity [22]. For example, increases in the firing rate of balanced pre-synaptic activity afferent to a neuron can reduce single neuron firing rate gain [27], [28], [29], [30], [31], [32]. Further, an increase in the temporal correlation between the arrival times of excitatory pre-synaptic inputs increases the firing rate of a post-synaptic target neuron [33], [34], [35], while correlations between excitatory and inhibitory inputs can reduce output activity [34], [36]. The impact of such shifts in the temporal structure of synaptic input is amplified when the post-synaptic cell has a small integration timescale, as expected for neurons in the high input rate, balanced state [22]. These examples deal with synaptic activity convergent to a single target cell. However, what is less studied is the role that the balanced state plays in modulating the responses of a pair of neurons subject to a common synaptic input. In this study, we consider this latter scenario and show that shifts in balanced pre-synaptic population activity modulate the magnitude and timescale of the correlations of spike trains from pairs of post-synaptic neurons.

We first explore a model system and show that output spike train correlations from a pair of neurons are modulated by varying the rate of uctuating, balanced excitatory and inhibitory inputs. Specifically, we demonstrate that an increase synaptic input rate leads to an increase of short-timescale output correlation (i.e. precise spike synchrony) while correlation at long timescales (i.e firing rate co-variation) remains unaffected, or even decreases. Due to the differential affects of our mechanism on short and long timescale spiking activity we label the combined modulation *correlation shaping*. Correlation shaping has been observed in various sensory systems [2], [3], [37], [38], yet the core mechanisms underlying the modulation remain unknown. We present linear response analysis showing that the enhancement of output synchrony through an increase of input rate results from a shift in single neuron integration properties that favors the transfer of high frequency inputs. Dynamic clamp recordings from cortical neurons verify our theoretical predictions. Finally, in a feedforward network model, we show how correlation shaping supports a selective propagation of network responses, so that activity can be gated by correlations in complex neuronal networks. In total, our work extends mechanisms of single neuron firing rate control include the control of pairwise correlations, thereby providing a bridge between single neuron and network state modulation.

## Methods

### Conductance-Based Neuron Model

We modeled neurons as leaky integrate-and-fire units receiving conductance input [39]. Each neuron had an intrinsic timescale 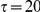 ms and leak reversal potential 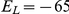 mV. Excitatory and inhibitory synaptic input caused conductance changes 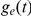 and 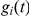 with reversal potentials 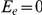 mV and 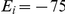 mV so that the membrane potential dynamics followed:
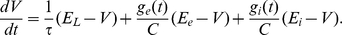
When 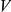 reached a threshold voltage 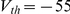 mV, the neuron spiked and the voltage was reset to 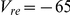 mV.

We modeled the excitatory and inhibitory synaptic conductances as Poisson processes with rates 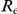 and 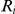 consisting of series of 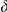-functions with heights 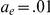 and 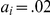. This framework was used for all of the simulations presented and provides a minimal model that captures our main results (for simulations of other models, see Supplementary Figures). These inputs consisted of independent processes private to each neuron as well as a shared component presynaptic to all neurons, yielding 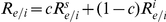 where superscripts 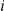 and 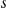 denote independent and shared components, respectively. For large rates, this input was approximated as a diffusion process [39], [40], [41], [42](Figure S1):
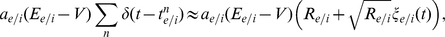
where 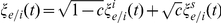 was a Gaussian white noise process with unit intensity. This allowed us to write our voltage equation in the form
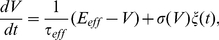
(1)where 
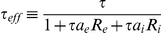
, 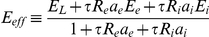, and 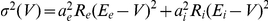. Note that as the rates of excitation and inhibition 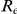 and 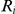 increase in a balanced manner, 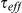 decreases, 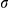 increases, and 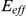 does not change substantially because of the excitation and inhibition balance.

For our simulations and calculations, we set 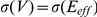. This approximation ignored the multiplicative nature of the noise, which in our simulations did not substantially change the results (Figure S1), since the change in 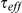 and 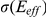 were sufficient to modulate neuronal responses. To simulate pairs of neurons receiving correlated input, we set the fluctuating input to each neuron to be
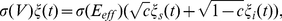
(2)where 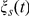 was shared across both neurons while 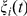 was independent for each neuron. We note that, although the correlation in output spike trains depended on the degree of pre-synaptic overlap, Eq. (2) shows that 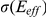, and hence the firing rate of neurons in our model, was independent of 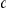. The rate of excitatory input in the low state was 1.50 kHz and 6.16 kHz in the high state, with the inhibitory rate chosen to elicit a firing rate of 15 Hz in both cases. Simulations were performed using an Euler-Maruyama numerical integration scheme with a simulation timestep of 0.005 ms.

### Solving for Transfer Function and Power Spectrum with Fokker-Planck Techniques

We next developed a theoretical framework to study the behavior of the above system and compared our theory against simulations of the stochastic system. For completeness, we write the governing equations used to calculate the single neuron power spectrum 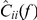 and transfer function 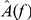; these techniques are fully presented in [42] and we refer the reader there for further details. Letting 
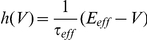
, the voltage distribution 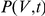 associated with the stochastic differential equation (1) obeys the Fokker-Planck equation:
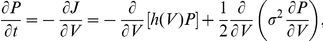
where 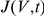 is the probability flux [43]. The boundary conditions for the probability distribution and flux at threshold are 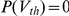 and 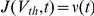, where 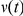 is the firing rate. Furthermore, the flux obeys 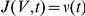 for 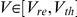 and is 0 otherwise.

For time independent 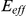 and 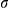 the steady state distribution 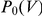 obeys:
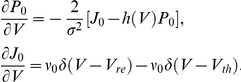
Using the normalization condition 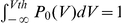, we can solve for the steady state firing rate 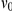.

In order to study the system's response to a correlated, fluctuating input, it is necessary to study the system's response to time-dependent inputs. This is done most effectively by writing a time-dependent Fokker-Planck equation in the Fourier domain:
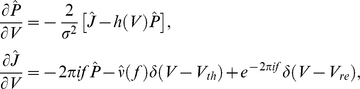
where (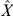) denotes the Fourier transform of 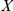 and 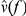 is computed with initial condition 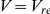. Solving this equation yields the Fourier transform of the first passage time density 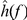 [42]. The power spectrum 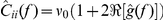, where 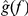 is calculated from the well known renewal relation 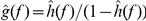 [44].

Finally, we compute the transfer function 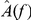. Suppose that we add a time-varying periodic current 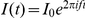 to the right hand side of Eq (1). If we let 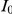 be sufficiently small, we can compute the spike train response to these time-dependent modulations. Decomposing the probability density, flux, and firing rate into steady state and modulated components:
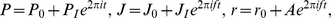
and then solving the Fokker-Planck equation for the time-dependent terms, we obtain a new set of equations:
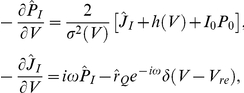
with boundary conditions
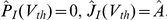
These equations were solved numerically [42] obtaining a solution for the transfer function 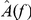.

### Experimental Techniques

Surgery: Somatosensory (S1) cortical slices were prepared from CBJ/Bl6 mice age P19-26. All surgical procedures followed the guidelines approved by the Carnegie Mellon Animal Welfare Committee. The mice were anesthetized with isoflourane and decapitated. The brain was exposed, removed from the skull and immersed, in ice cold oxygenated (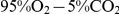) ACSF (in mM: 125 NaCl, 2.5 KCl, 25 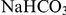, 1.25 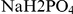, 1.0 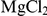, 25 Dextrose, 2 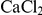) (all chemicals from Sigma, USA). Coronal slices (300 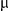
m) of barrel cortex made using a vibratome (Leica, Place). The slices were maintained in ACSF at 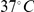 for 30 min then rested at room temperature (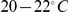) for 1 hr prior to recording (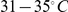).

Electrophysiology: L2/3 pyramidal neurons were visualized using infrared-differential interference contrast microscopy (Olympus, Center Valley, PA). Whole cell, dynamic clamp recordings were performed using a MultiClamp 700B amplifier (Molecular Devices, Union City, CA). Data were low pass filtered (4 kHz) and digitized at 50 kHz using an ITC-18 (Instrutech, Mineola, NY) controlled by custom dynamic clamp software (R. Gerkin; http://rick.gerk.in/software/recording-artist/) written in IgorPro (Wavemetrics, Lake Oswego, OR). Pipettes were pulled from borosilicate glass (2.0 mm, outer diameter) on a Flaming/Brown micropipette puller (Sutter Instruments, Novato, CA) to a resistance of 6–10 M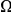. The intracellular solution consisted of (in mM) 130 K-gluconate, 5 KCl, 2 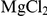, 4 ATP-Mg, 0.3 GTP, 10 HEPES, and 10 phosphocreatine.

Stimulation: Pyramidal cells (n = 8) were directly stimulated by a series (50–100 trials) of simulated noisy synaptic currents in dynamic clamp. Each trial was 4 s in duration with a 5 s inter-trial interval; the period of rest was used to ensure that stability of the recordings. For each trial, excitatory (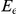: 0 mV) or inhibitory (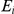: −60 mV) synaptic conductance inputs were simulated as Poisson distributed spike times convolved with alpha function 
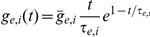
. (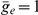 nS, 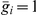 nS, 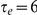 ms, 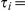
8 ms). The Poisson rates for excitatory and inhibitory inputs were equal to one another (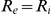), and were set to 3 kHz in the low state and 7.5 kHz in the high state. These rates were higher than in the simulations to ensure high spike time variability, since the input variability is attenuated by the finite temporal extent of the synaptic timescales. For each state, half of these inputs were common to all neurons stimulated and half were newly generated on each trial for every neuron. This produced an input correlation, 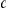, of 0.5 between any given pair of neurons. This setup permitted 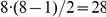 pairwise comparisons. Since the synaptic drive was subthreshold, a bias current (0.3–0.7 nA) was added such that the balanced conductance fluctuations produced a mean cortical firing rate of (4–6 Hz) in both the low and high states.

### Feedforward Network

We studied a layered network in which a population of 100 leaky integrate-and-fire neurons (Layer 2) received balanced input from a pre-synaptic layer (Layer 1) with 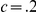 and provided excitatory input to two distinct downstream targets. Neurons in Layer 1 were assumed to be Poisson as in previous sections, and the total input to a Layer 2 neuron was therefore approximated by a diffusion process. In particular, the voltage dynamics of each Layer 2 neuron followed Eqs. 1 and 2.

The downstream target was also modeled as leaky integrate-and-fire neuron. Because we wished to fix the timescale of the downstream target, we assumed delta-function, current-based synapses so that the voltage 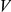 of the downstream neuron followed:
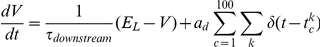
where 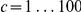 indexes the neurons in Layer 2 and 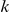 indexes the spikes in each Layer 2 neurons' spike train. We compared 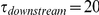 ms and 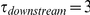 ms. For 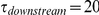 ms, we set 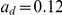 mV and for 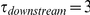 ms, 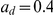 mV so that the neurons fired at comparable rates given identical input. Other parameters, including leak, threshold, and reset voltages were identical to the model previously studied.

## Results

### Modulation of Correlation Susceptibility

In general, it is difficult to determine the specific changes in a neural system's dynamics that cause changes in spike train correlations. We studied a framework in which common inputs drive the correlations between the spike trains of a pair of neurons [45], [46], [47]. If the degree of input correlation, 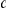, is small, a linear approximation relating 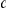 to the output spike correlation, 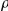, is written as:
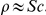
Here the quantity 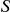, termed the *correlation susceptibility*, determines the extent to which two neurons' spike trains will be correlated given a fixed level of correlation between the inputs they receive [17].

**Figure 1:**
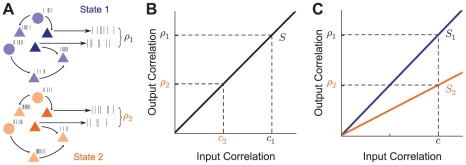
Mechanisms of correlation modulation. (A) The spike train correlation between a pair of neurons shifts from 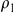 to 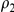 as the state of pre-synaptic field shifts. (B) Mapping between input correlation 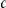 and output correlation 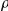. The change in output correlation in panel A may be due to a change in input correlation from 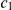 in state 1 to 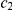 in state 2. (C) An alternative mechanism by which output correlation can change is that the correlation susceptibility 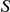 changes from 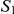 in state 1 to 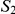 in state 2, with input correlation 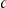 fixed throughout.

Throughout this study, we focused on a pair of neurons that shift their output correlation (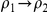) due to a change in their pre-synaptic drive (Figure 1A). Under our linear model, two simple explanations for the shift in output correlation are possible. First, the shift may simply reflect a change in the correlation of the inputs that the neuron pair receives (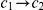; Figure 1B). While this answer appears straightforward, understanding shifts in input correlation requires detailed anatomical knowledge of the network architecture, in the absence of which simplifying assumptions are required [48].

A second explanation for the shift in output correlation is a shift in correlation susceptibility (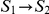), even when the input correlation remains fixed (Figure 1C). Because 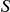 relates the correlations in the spiking output of neurons to their common input, we expect 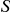 to be sensitive to how each neuron integrates its input. Indeed, single neuron response properties such as firing rate and neural excitability determine the extent to which neurons become synchronized by shared input [49], [17], [18], [19], [20]. There has been substantial work on how single neuron properties, such as firing rates, are modulated [27], [28], [29], [30], [31], [32], [50], [51], [52], [53], [54],suggesting that 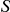 should also be open to modulation. We focused on this second mechanism and established how modulations of single neuron responses also modulated pairwise correlations in cortical populations.

### Low and High Rate Synaptic Input States

**Figure 2:**
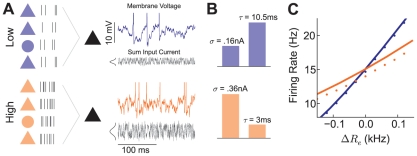
Single cell statistics in low and high synaptic input states. (A) Left: Schematic of low (top) and high (bottom) states. Excitatory inputs occurred at a rate of 1.5 kHz in the low state and 6.2 kHz in the high state, modeling the activity from a pool of pre-synaptic cells. Inhibitory inputs were chosen so that output firing rates were fixed at 15 Hz in both states. Right: Synaptic inputs converged onto a conductance-based leaky integrate-and-fire neuron model. Sample membrane potential traces of the neuron model in both the low (top) or high (bottom) states are shown. The total input current in either state is plotted below each membrane potential trace. (B) The input current variability (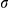) and membrane potential time constant (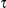) for both the low (top) and high (bottom) input states. (C) Firing rate of a neuron as the level of excitatory input is varied, showing decreased gain in the high input state compared to the low input state. The balanced condition in both low and high states resulted in an output firing rate of 15 Hz. Solid lines were calculated using our theory (see Methods). Dots correspond to numerical simulations of the model system. Standard error is smaller than the width of the dots.

We first investigated the transfer of input correlations to output spike train correlations in a simplified two-neuron network. Each neuron received conductance-based, pre-synaptic inputs from a mixed population of excitatory and inhibitory neurons (Figure 2A). To model the stochastic nature of cortical activity, the arrival times of both excitation and inhibition were modeled as Poisson processes. We set the relative strengths and rates of excitation and inhibition so that the mean input was balanced [23], [24], and the average membrane potential was below spiking threshold. Balanced pre-synaptic activity results in large membrane fluctuations that trigger spikes in a random, aperiodic pattern, consistent with *in vivo* recordings from cortical neurons [21], [22].

Shifts in the activity level of a recurrent cortical population are observed in many neural systems and have been shown to affect the response properties of neurons *in vitro* and *in vitro* [22], [55], [31]. To explore the modulatory effects of balanced synaptic input, we considered the neuron model in two states: a *low* state, in which pre-synaptic input arrived at a low rate, and a *high* state, in which pre-synaptic input arrived at a high rate (Figure 2A). While the level of balanced fluctuations may lie on a continuum, we compared two representative points, analogous to high and low activity states in a cortical network [56], [26]. A clear consequence of the shift from low to high states was an increase in the variability of both the input current and membrane potential response, due to greater fluctuating input (Figure 2B). This increase of input variability was reflected in an increase in spiking variability, with the coefficient of variation of the inter-spike intervals increasing from 0.73 in the low state to 0.91 in the high state. A second consequence of an increase in pre-synaptic rate was the reduction of the membrane time constant 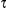 (Figure 2B). This was expected, since the membrane time constant 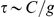, with 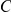 the membrane capacitance and 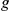 the total membrane conductance [57]. As 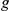 is roughly proportional to the pre-synaptic rates, an increase in the rate of synaptic input lead to a decrease in 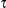. Taken together, the shift from the low to high state evoked a more stochastic and faster membrane potential response.

We first examined the effect of balanced synaptic input on firing rate gain, the slope of the firing rate curve when plotted as a function of excitatory input strength. When the rate of balanced excitatory and inhibitory synaptic input changed from low to high, the neuron's firing rate gain was substantially reduced (Figure 2C). This gain decrease in the high background state has been studied extensively in theoretical and *in vitro* work [27], [28], [29], [30], [31], [32] as well *in vivo* under specific stimuli conditiona [31]. In the high state,larger membrane potential fluctuations increased firing rates for weak inputs. However, there was also a decrease of the net membrane input resistance, causing an increase in the rheobase current (minimum steady current required to recruit spiking). The combination of these two effects lead to an overall reduction in firing rate gain [29]. We next explored the consequences of gain modulation via balanced activity for correlation transfer by pairs of neurons.

### Correlation Shaping with Synaptic Activity

**Figure 3:**
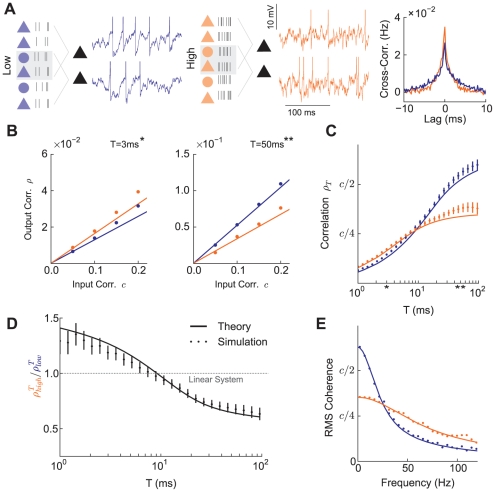
Pairwise cell statistics in low and high rate synaptic input regimes. (A) Schematic of low (left) and high (center) states with sample membrane traces. The marginal statistics of both cells are as reported in Figure 2, with a fixed overlap of excitatory and inhibitory pre-synaptic inputs for the cell pair. The input correlation is 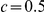 for membrane traces and 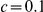 otherwise, in both low and high states. Right: Spike train cross-correlation functions for the firing of the two neurons when receiving correlated input, showing state dependent shaping. (B) Relationship between spike count correlation 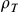 for windows of length 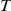 and input correlation 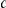, showing linearity for small 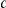 and a dependence on 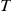. (C) Output correlation as a function of window size in the high and low states. Asterisks mark the values of 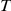 that correspond to the plots in Figure 3B. (D) Ratio of correlations as a function of window size in the high and low states, showing favoring of short timescale synchrony in the high state. For comparison, the lack of correlation shaping for a purely linear neural transfer is indicated. (E) RMS coherence (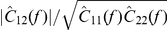) between spike trains showing a decrease in low-frequency coherence and increase in high frequency coherence in the high state. The theoretical results (solid lines) shown in in panels (B) through (E) were derived from a linear response calculation valid in the small 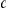 limit (see Methods). Bars denote standard error in (B) through (D). In (B), standard error is smaller than the width of the dots.

To study the effects of balanced excitatory and inhibitory inputs on pairwise spike train correlations, we extended our model to include a pair of post-synaptic neurons receiving overlapping pre-synaptic inputs (Figure 3A). Previous work has shown that the output firing rate affects correlation susceptibility [17]. To preclude any firing rate-induced effects, the synaptic input was adjusted so that the average output firing rate of each neuron remained at 15 Hz in low and high states (Figure 2C). Furthermore, there was a fixed overlap in the input populations, so that the input correlation also remained constant in both network states (Figure 3A). Thus, any change in the output spike train correlation induced by changing synaptic input will be due exclusively to a shift in correlation susceptibility (Figure 1C).

We found that the timescale over which the two spike trains were correlated was dependent on the level of balanced synaptic activity (Figure 3A, Right). When the synaptic rate increased from the low to high state, the magnitude of the peak of the cross-correlation function near zero lag increased, reflecting greater spike time synchrony between the neurons. However, this increase was not present for longer lags, and the spike train cross-correlation function was unchanged or reduced for sufficiently long lags (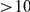 ms).

To quantify this change in output correlation over a range of timescales, we first counted the number of spikes 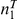 and 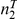 that the two neurons emitted in intervals of 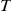 milliseconds. We next computed the spike count correlation as a function of window size:
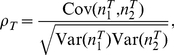
(3)where Cov and Var denote covariance and variance, respectively. In the framework of our simple circuit (Figure 3A), correlation in output spike trains 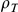 was a consequence of a shared input correlation 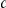. For small 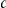, linear response theory [17] takes the output correlation to be a linear function of the input correlation (Figures 1B,C; 3B):
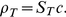
(4)In our model, this linear relationship held for a range of 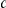, in both low and high states and at both short and long 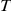 (Figure 3B). Further, the 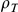 values produced were, in magnitude, consistent with *in vivo* recordings from a variety of systems [58], [3], [2], [6]. When comparing 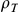 for the low and high states at fixed 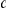, a differential change of correlation at different timescales was evident. Specifically, 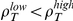 for small 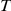 (Figure 3B, 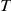 = 3 ms), while 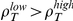 for large 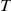 (Figure 3B, 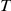 = 50 ms). This differential modulation of correlation occured over a broad range of timescales, with 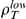 and 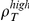 intersecting only once (Figure 3C), and we label the modulation a *shaping* of correlation [38]. This substantial change in both the magnitude and timescale of correlation must involve a nontrivial change in how the neurons process their inputs, since the input correlation 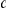 and firing rate were the same in both low and high states. We note that the qualitative results of our study are also valid for larger 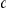 (Figure S2) and different synaptic strengths (Figure S3).

Since 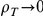 as 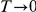 [59], changes in 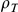 at small 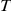 are necessarily smaller in magnitude. However, synchrony at short timescales can have large effects on downstream targets sensitive to coincident pre-synaptic spikes [12] and indeed the peak of the cross-correlation function increased substantially in the high state (Figure 3A, Right). To properly compare correlation shaping at small and large 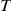 we considered the ratio 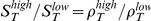, providing a relative measure across the low and high states. The ratio was a decreasing function of 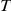, with substantial changes in correlation at both short and long timescales (Figure 3D). The negative slope of the curve indicates that increases in the rate of balanced synaptic activity favor spike synchronization rather than long timescale correlation. Finally, the spectral measure of spike train coherence between the two spike trains in both states exhibited a decrease for low frequencies but a significant increase for high frequencies in the high state (Figure 3E). Here, the increase for high frequencies, which occurs over a broad range of frequency space, is related to the increase in short timescale synchrony, consistent with the spike count correlation shaping.

Correlation shaping is an unexpected feature of balanced synaptic activity. For subthreshold membrane potential dynamics (or any other linear system) the ratio 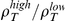 is equal to 1 for all 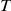 assuming a fixed input correlation (Figure 3D, gray line). The mechanism that shapes correlation transfer so to promote spike train synchronization over long timescale correlation in the high state (Figure 3D) is the focus of the next section.

### Relationship between Correlation Susceptibility and Neuronal Integration

Correlation shaping is a property of the joint statistics of a pair of neurons. However, since the input correlation was the same in the low and high states of our model, then the mechanism underlying the shaping is hypothesized to be related to changes in single neuron input integration and spike emission across the two synaptic states (Figure 1C rather than 1B). In this section, we show that correlation shaping is a consequence of a shift in the single neurons' frequency response across the low to the high input state.

The spike train auto-correlation 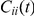 and cross-correlation 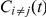 functions are written as:
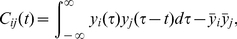
(5)where 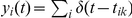, with 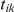 labeling the 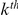 spike time from neuron 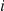 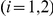. Here 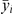 is the mean firing rate of neuron 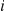. We are interested in the joint spike count correlation for the neuron pair, where the spike count for neuron 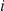 over a window of length 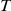 is 
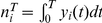
 (we take the neuron's stochastic dynamics to be in statistical equilibrium). The spike count variance and covariance are related to integrals of auto- and cross-correlation functions [44], yielding an alternate expresion for 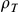:
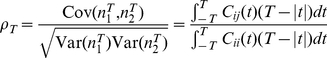
(6)In the second equality we have, for simplicity, assumed that 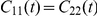 (or equivalently 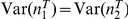). These integrals can be transformed to the frequency domain, using the Wiener-Khinchin theorem [44] to relate correlation functions 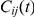 to their spectral analogues 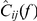, yielding
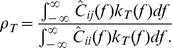
(7)Here 
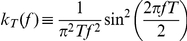
 is the Fourier transform of the triangular weighting term in Eq. (6). Our strategy was to relate the cross spectrum between the spike trains, 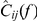, to single neuron integration properties.

**Figure 4:**
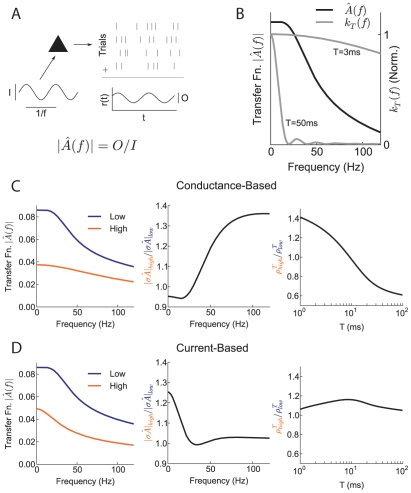
Relating correlation shaping to single neuron transfer (A) Illustration of neuronal transfer function. A perturbing input of amplitude 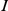 and frequency 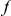 causes a modulation of the spike response of a fluctuation driven neuron. Averaging across stimulus presentation trials gives the average output firing rate 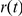 with amplitude 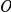. The output-input ratio defines the neural transfer 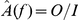. (B) Example 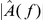 for a fluctuation driven neuron (black curve). The weighting function 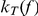 for 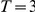 ms and 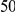 ms (grey curves). (C) Left: transfer function 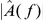 for neurons in the low and high background states. Center: Ratio of transfer functions in the two states (normalized by change in input strength 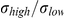). Right: Ratio of correlations as in Figure 3D. (D) Same as (C), but for a current-based model in which 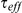 does not change in the high state. Note correlation shaping in (C), Right but not (D), Right.

Single neuron input-output transfer is typically expressed through its spectral transfer function 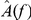. The transfer function measures the ratio of the amplitudes of a neuron's firing rate response and a small amplitude sinusoidal signal of frequency 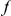 (Figure 4A). For very slow inputs, the transfer function 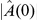 equals the firing rate gain, since this measures the sensitivity of firing responses to static (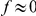) inputs. For 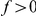, 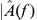 is the susceptibility for a neuron's trial averaged response to be locked to a time varying signal. The transfer function 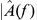 is experimentally measurable [60], and is related to the more commonly reported spike triggered average [61]. In general, for neurons in the fluctuation-driven regime, 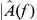 is a decaying function of 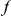 (Figure 4B).

If each neuron receives a small shared signal 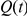, then we can write the expectation of the Fourier transform of the spike train from neuron 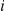 as:
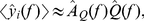
(8)where the brackets denote an average over repeated frozen presentations of the shared signal 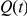 with different realizations of the independent noise driving the neurons [62]. Here, 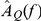 is the linear response of the system to the perturbation 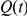. Finally, averaging the quantity 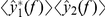 over different realizations of the process 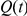 yields the cross-spectrum between neurons 1 and [17], [62], [63], [64]:
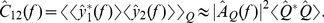
(9)For the case of white noise input, we have that 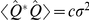. With Eqs. (7) and (9) we calculated the spike count correlation coefficient between the two neurons receiving shared white noise input as
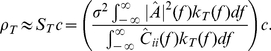
(10)Our theory then relates single neuron transfer 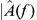 and power spectrum 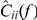 to the joint pairwise response 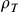.

The theoretical predictions given in Eq. (10) gave a very good quantitative match to simulations of the leaky integrate-and-fire neuron pair (Figures 3B–E, compare solid curves to points), capturing the correlation shaping between the two states. Eq. (10) has been previously derived [17], [18], however, the model neurons considered in those studies were current driven model neurons. We considered conductance driven model neurons, meaning that the calculation of 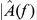 and 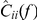 must account for the linked shifts of the membrane time constant and membrane potential fluctuations from the low to the high state (Figure 2B). For our conductance based integrate-and-fire model neurons, the quantities 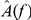 and 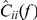 were calculated by numerically integrating the Fokker-Planck equation associated with the stochastic differential equation expressed in Eq. (1) (see [42] and Methods). The distinction between current and conductance based neural integration will be shown to be critical for correlation shaping.

Before correlation shaping is related to the shifts in 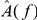 between the low and high states, we first discuss the dependence of susceptibility 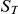 on the window size 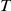 (Figure 3B). This dependence enters equation (Eq. (10)) through the weighting term 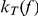, which determines the contribution of 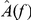 across frequency to 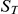. For long timescales (large 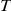), 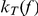 is low-pass, so that only the neurons' response to low frequencies contributes to correlation susceptibility. In contrast, for short timescales (small 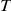), 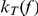 weighs the transfer function approximately equally across all frequencies. Hence, the neurons' high frequency response determines precise spike synchrony. Indeed, for 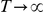 we have that 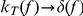, while 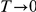 limits 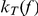 to a constant function on 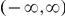. Therefore, for large 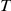, only the zero-frequency components of 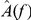 contribute to the integral, while for small 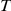, all frequencies contribute.

A mechanistic understanding of correlation shaping (Figure 3D) requires knowledge of how the rate of balanced synaptic activity affects the transfer function. As discussed previously, the increase in synaptic input from the low to the high state decreased the effective membrane time constant of the neuron 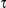 while it increased the input variability 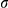 (Figure 2B). The decrease in 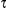 corresponded to a decrease in the timescale over which a neuron integrates inputs and hence an attenuation of the neuron's transfer function. For low frequency inputs, this reduction was precisely the firing rate gain control known to occur with increased synaptic input (Figure 2C). Increased variability and shunting due to heightened conductance reduced the neuron's ability to respond to slow depolarizing inputs. However, the reduction in the transfer function from the low to high state was not uniform across all frequencies (Figure 4C, Left). This was because the smaller value of 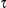 in the high state enhanced the tracking of fast inputs, mitigating the attenuation of the transfer function for high frequencies. The combination of the non-uniform attenuation of the transfer function and increase in 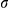 from the low to high state determined the shaping of the correlation susceptibility 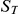 (see Eq. (10)).

To illustrate the shift in single neuron response between the low and high states, we considered the quantity 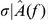, the strength of the input fluctuations multiplied by the input transfer function. The ratio 
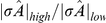
 was an increasing function of frequency (Figure 4C, Center), indicating that high frequency transfer is favored in the high state. In general, a favoring of high frequencies corresponds to a favoring of synchrony, measured over only small 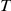 (since 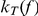 is nearly flat across 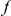 for small 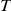). Thus, the high state is expected to favor small 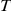 correlation transfer compared to the low state (Figure 4C, Right). In contrast, for large 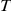 which corresponds to low frequencies, correlation transfer was disfavored in the high state (since 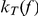 only weights low 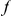 for large 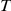). This ratio allowed us to intuitively link correlation shaping over different timescales to the shaping of the transfer function over different frequencies.

We argue above that a change in the effective membrane time constant is central to the correlation shaping we discuss. To demonstrate this fact, we computed the transfer function and correlations for a current-based model in which 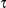 remained unchanged in the low and high state, although 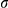 increased by the same amount. If firing rates were again fixed at 15 Hz, the transfer function was again reduced in the high state, but the ratio 
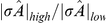
 remained close to unity (Figure 4D, Left and Center). As a result, no substantial correlation shaping was observed (Figure 4D, Right). The above comparison shows that this shaping requires the modulation of cellular properties that is allowed by a conductance-based model.

Finally, we note that, although our analysis has focused on the numerator of Eq. (7), the denominator also affects the correlation for large time windows (Figure S4). For these values of 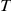, the denominator was increased in the high state, reflecting the higher variability of firing due to stronger input fluctuations. This further attenuated the value of 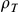 for large 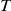 in the high state.

### Correlation Shaping with Different Output Firing Rates

To avoid changes in correlation owing to firing rate [17], we chose the balance between excitation and inhibition in previous sections so that firing rate was fixed across both the low and high states (Figure 2C). However, it is unlikely that firing rates will remain fixed as a network shifts from a low conductance to a high conductance state. Thus, it is important to understand how correlation shaping via balanced excitatory and inhibitory inputs interacts with the correlation changes expected due to firing rate changes. In this section we show how the modulations of correlation due to balanced excitatory and inhibitory inputs and those due to firing rate changes from imbalanced inputs are distinct.

**Figure 5:**
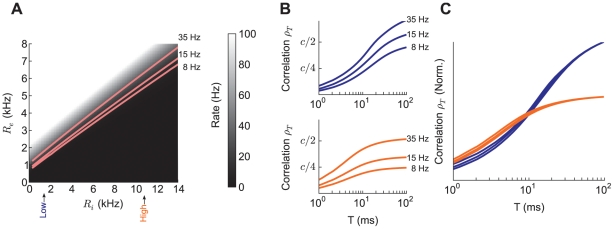
Comparing correlation shaping due to balanced excitatory and inhibitory inputs and correlation shifts due to shift in output rate. (A) Output firing rate as a function of 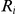 and 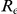 (firing rates above 100 Hz not shown). The curves in the space that yield output firing rates of 8,15, and 35 Hz are labeled. The lines lie in a small region of the full space, corresponding to the region where excitation and inhibition are balanced. (B) Top: Spike count correlation as a function of 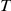 for the three output rates, where rate changes are due to a change 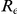, with 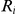 fixed at the low state value. Bottom: same as top, except that 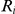 is fixed at the high state value. (C) The curves in B for the low and high states scaled to match the center curve (15 Hz) at 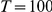 ms.

The firing rates of our output neurons were determined by the input rate of both the excitatory (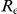) and inhibitory (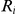) inputs. In fact, for any desired output rate, there was a curve in (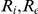) space that achieved that rate (Figure 5A). For moderate input rates, a balanced shift in input (approximately linear in 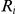 and 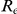) preserved output firing rate. A change in output firing rate (switching from one curve to another in Figure 5A), can occur from a shift in 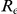, a shift in 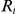, or some combination of the two. When we fixed 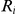 to its value in the low state and increase 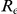 so that the output rate increased, 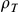 increased over all timescales 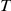 (Figure 5B, top), as expected [17]. A similar effect occured if we repeat this in the high state (Figure 5B, bottom). Thus, the modulation of 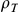 by a rate change due to an imbalanced shift of 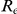 simply scales 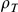 for all 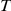 (collapsed blue and orange curves in Figure 5C). Nevertheless, after correcting for the rate scaling of 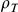, the shaping of correlation between the low and high states remained clear (Figure 5C), demonstrating that correlation shaping due to a change from low to high states is distinct from correlation shifts due to arbitrary output firing rate changes.

To illustrate this, we considered a shift from 8 Hz in the low state to 35 Hz in the high state. In the shift from the low to high state, the effective membrane timescale 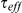 shifted from 10.8 to 2.9 ms and the amplitude of the input fluctuations 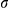 from 0.16 to 0.37 nA. These shifts changed 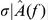 significantly (as discussed in the previous section), and changed the timescales over which the neuron pair was correlated. This was contrasted by a shift from 8 Hz to 35 Hz in the low state: a change in firing rate without a change between low and high states. Here, 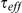 shifted from 10.8 to 10.2 ms and the input fluctuations 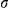 from .16 to .18 nA, having little influence on 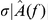 other than a uniform scaling due to the output rate change. In total, by changing both 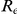 and 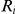, it was possible to not only change the output firing rate so as to amplify or attenuate 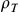, but also to shape the timescales over which a neuron pair was correlated.

### Experimental Verification with Dynamic Clamp Recordings

**Figure 6:**
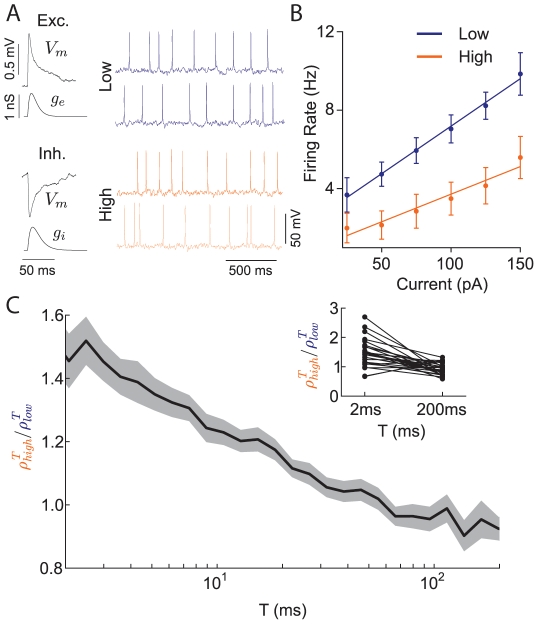
Correlation shaping in cortical dynamic clamp experiments. (A) Left: Recorded average EPSPs and IPSPs from resting neurons showing membrane voltage (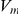) deflections, with corresponding conductances 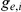. Right: Voltage traces from example recorded neuron pairs in high and low states. The degree of synaptic overlap was 0.5 for both high and low states. The inter-spike interval coefficient of variation increased from 0.40 in the low state to 0.48 in the high state. (B) Firing rate versus mean input current curves for neurons in low and high states showing reduction in gain in the high background state. (C) The ratio of 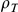 in the high to low state as a function of window size 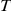 (compare to Figure 3D). Curves are population average results (n = 28) with the shaded region denoting the standard error. Inset: Correlation ratio shown at 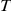 = 2 ms and 200 ms for each recorded pair.

Our two-neuron framework for studying correlation transfer (Figure 1A) permited an experimental verification of correlation shaping with balanced, fluctuating conductance inputs. We performed *in vitro* patch clamp recordings from cortical pyramidal neurons receiving simulated excitatory and inhibitory inputs. Unlike past experimental studies of correlation transfer [16], [17], our model involved conductance-based, rather than current-based synapses. Therefore, we simulated synaptic input using dynamic clamp [65] (see Methods), which affected the membrane integration timescale as well as membrane potential variability. We chose maximal excitatory and inhibitory conductances of 1 nS and synaptic timescales of 6 and 8 ms, respectively, producing a synaptic input that was more biophysically realistic than the diffusion process used in previous sections (Figure 6A). The shift from low to high state caused a near two-fold reduction in firing rate gain (Figure 6B), in qualitative agreement with our model simulations (Figure 2C) and past dynamic clamp studies [29]. Further, as was done in the model, we set the synaptic balance in the low and high states to produce approximately the same firing rate (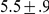 Hz in the low state and 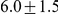 Hz in the high state).

The correlated input for a given neuron pair was a mixture of shared and independent excitatory and inhibitory inputs, mimicking the input provided to the model (Figure 3A). The partial overlap in the synaptic input produced correlated membrane potential and spike dynamics for every neuron pair in both the low and high states. Our recorded spike trains showed a dependence of spike count correlation on 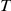 that was qualitatively similar to that of the model, apparent in the ratio of 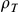 in the high and low states (Figure 6C). The ratio was a decreasing function of 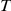, indicating a bias toward synchrony in the high state compared to the low state. This shape was consistent with our model results (Figure 3D), although the ratio did not fall substantially below unity in the limit of large 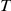. This suggested that the decrease in gain 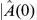 and the increase in variability 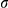 from the low to high state were of similar magnitudes, since in the limit of large 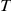 correlation susceptibility is proportional to 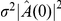 (Methods). A conductance-based simulation using the same synaptic parameters used for dynamic clamp stimulation produced results in agreement with the experiment (Figure S5). The favoring of synchrony (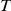 = 2 ms) over long timescale correlation (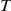 = 200 ms) in the high state was statistically significant in a pairwise analysis across the dataset (Figure 6C, inset; 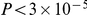, paired *t*-test). The experiments demonstrated that an increase in the rate of balanced conductance input shapes pairwise correlation so as to favor synchronization over long timescale correlation, thereby verifying the main theoretical predictions of our study.

Our theoretical treatment has ignored the timescale of synaptic input, and has associated all filtering to the membrane and spike properties of the model (Figure 4). Correlation transfer with realistic synaptic timescales did quantitatively differ from the case with instantaneous synaptic input (Figure S5B). Nevertheless, our theoretical work captured the main effects of correlation shaping when synaptic timescales were realistic (Figures 6 and S5). This is because only the effective membrane time constant was sensitive to a shift in input firing rate, which our theory accounts for, while synaptic filtering did not change between low and high states. We remark that, for synapses with very long timescales, correlation shaping should only be present for large 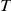, since correlations at small 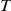 will be negligible.

### Consequences of Correlation Shaping for Signal Propagation

The spike train correlations between neuron pairs substantially influence the propagation of neural activity in feedforward architectures [13]. For example, while our study has so far focused on the transfer of correlation for neuron pairs receiving common input, the firing rate of a single downstream neuron also depends on the correlation between neurons in its pre-synaptic pool [12]. If the integration timescale of the downstream target is small, only precise spike synchrony will effectively drive the neuron. In contrast, neurons that slowly integrate inputs will be sensitive to long timescale correlations. In our study, we demonstrated that an increase in the rate of synaptic input increases spike count correlation at small 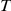 while simultaneously decreasing the correlation at large 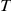 (Figure 3D). We therefore expected that this correlation shaping would influence the extent to which activity can be propagated to a downstream layer. Further, that the magnitude of this effect would depend on the integration timescales of the downstream targets.

**Figure 7:**
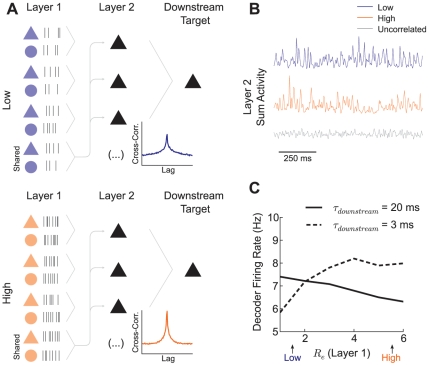
Effects of correlation shaping on the propagation of neural activity. (A) Schematic of layered network. Layer 1 neurons are modeled as Poisson processes and are either in the low (top) or high (bottom) state. Each layer 2 neuron receives a combination of private input and a globally common input from layer 1. The common input correlates each pair of layer 2 neurons, while the state of layer 1 shapes the correlations (cross correlation function insets). The layer 2 neurons have marginal and pairwise statistics identical to the neurons in Figures 2 and 3. The spike outputs of the layer 2 neurons converge onto a downstream neuron with integration timescale 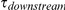. (B) Example realization of the summed synaptic activity that drives the downstream target neuron in the low (top), high (middle), and, for comparison, when the layer 2 neurons are uncorrelated (bottom). (C) Effect of the state change on the downstream neuron's firing rate. The horizontal axis 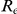 shows the level of excitatory synaptic activity that the neurons in the second layer received from the first layer. 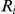 is adjusted in a balanced fashion so that the layer 2 neurons fire at 15 Hz (see Figure 5A). The downstream target neuron has either 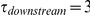 ms or 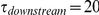 ms. The neuron with the fast time constant was driven more strongly in the high state. However, the neuron with the slow time constant showed a decreased firing rate in the high state.

As an illustration of this effect in a simplified system, we studied the firing rate of a downstream neuron receiving input from an upstream population of correlated neurons (Figure 7A; Methods). The level of synaptic drive from layer 1 shaped the correlation of pairs of layer 2 neurons (Figure 7A, insets). The network was constructed so that the activity of any given pair of neurons in Layer 2 was equivalent to that of the neuron pairs studied in previous sections. As the correlation of layer 2 spike outputs was shaped, so too was the magnitude and timescale of the synaptic drive to the downstream target neuron (Figure 7B). For comparison, we show that downstream target's synaptic input when the layer 2 neurons were uncorrelated (Figure 7B, bottom), showing significantly reduced variability [12]. In the uncorrelated case, the firing rate of the downstream target was much less than 1 Hz, indicating that correlated input was necessary for its recruitment.

We study how correlation shaping of the layer 2 projections affected the recruitment of the downstream target neuron. In particular, we focused on how the changing timescale of correlation recruited downstream targets differentially, depending on their own integration properties. We varied the rate of balanced synaptic input from layer 1 to layer 2 in a smooth manner (following the 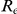 and 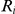 path for 15 Hz output in Figure 5A), gradually shaping the correlation function between any given layer 2 neuron pair. The shaping included the low and high states described earlier as near endpoints on a continuum (Figure 7C). When the downstream target had a smaller time constant (3 ms), its firing rate was increased when the pre-synaptic population was in the high state (Figure 7C, dashed line). This contrasted with the decreased firing rate in the high state when the downstream target had a longer time constant (20 ms) (Figure 7C, solid line). This differential effect was due to matching between the correlation timescale of layer 2 and the integration timescale of the downstream target. In the high state, synchrony drove the neuron with the short integration timescale, while, in the low state, long timescale correlations drove the slower neuron. Note that the firing rate of layer 2 neurons was unchanged in all cases studied. This simple example demonstrates that the structure of correlations between pre-synaptic neuron pairs can differentially drive downstream targets depending on their integration properties.

## Discussion

We have demonstrated that the rate of balanced synaptic input changes the correlation timescale of spike trains of a pair of neurons receiving partially correlated input. High rate synaptic input promoted precise spike time synchrony, while low rate synaptic input enhanced long timescale correlation. This correlation shaping was independent of changes in input correlation or the output firing rate of the neuron pair. Rather, it required a thresholding nonlinearity between input and spike train response as well as a state-dependent integration timescale. Both of these are properties of many neurons in the central nervous system, and hence we expect that similar correlation shaping may occur in a variety of brain regions.

### Correlation Shaping Compared to Other Forms of Correlation Modulation

Correlated neural activity continues to receive increasing attention [1], prompting investigations of the mechanisms that determine the transfer of correlation. Correlations are typically measured only at one timescale, but as we have shown, the magnitude of correlation depends on the timescale being considered, as does the likely significance of this correlation for activation of downstream neurons. Past studies have highlighted the dependence of spike train correlations on the magnitude of input correlation [15], [16], the form of spike excitability [19], [66], or the firing rate of the neuron pair [17], [18]. However, how the timescale of correlations are modulated through plausible mechanisms had not been addressed. Changes in membrane conductance have been widely studied and strongly influence the dynamics of single neuron activity [22]. In our study, we found that timescale-specific changes in neural correlations are a necessary consequence of conductance based modulation schemes. Previous work that has examined how correlated activity is transferred has used linear response methods to examine the response of neurons to current fluctuations, thereby leaving membrane integration invariant [17], [18]. As a result, cellular properties such as timescale were not modulated (see Figure 4D). We showed that when synaptic conductance is considered, it is possible to shape both the magnitude and timescale of output spike train correlations. This is a novel result that is nevertheless consistent with, and complementary to, the observation that firing rate also modulates correlations (see Figure 5).

### Noise Correlation Shaping in Neural Circuits

The widespread use of multi-unit recording techniques to study population activity has produced an increasingly clear picture of how neuronal spike trains are correlated in a variety of neural states. Recently, there has been particular interest in noise correlations, which are specific to within trial comparisons and cannot be directly attributed to a common signal [10].

Several groups have reported noise correlation measurements, ranging from small positive values [58], [2], [3], [6], [7], [8] to values that are, on average, zero, with positive and negative values equally represented [4], [67], [48]. Furthermore, in cases where significant noise correlation is measured, it can be modulated on distinct timescales. In the visual system, for example, noise correlation measured on timescales less than 100 ms is largest for cells with similar preferred stimulus orientations being driven at that orientation, observed in both spike responses [2] and synaptic input [37]. Further, while increasing stimulus contrast enhances short timescale correlation, it reduces long timescale (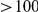 ms) correlation [2]. In primate area V4, stimulus attention reduces noise correlation when measured on timescales that are larger than 100 ms, yet has little influence on short timescale correlation [7], [8]. In contrast, other groups have shown that stimulus attention enhances spike synchrony measured at the gamma frequency timescale (20–40 ms) [68]. In the electrosensory system, long timescale noise correlation is reduced by recruitment of a non-classical receptive field, while synchrony is increased under the same conditions [3]. Thus, spike train noise correlations provide an excellent framework to study how the magnitude and timescale of correlations are shaped by neural state changes.

While a shaping of output correlation observed in these systems may be inherited from a state-dependence of input correlation (Figure 1B), single neuron response properties are often also modulated by network state. This suggests that a shift in correlation susceptibility may underlie a shift in pairwise correlation (as in Figure 1C). Indeed, firing rate gain is modulated by attention [69], stimulus contrast [21], and the recruitment of a non-classical receptive field [70]. In many cases, intracellular recordings have established that gain control is mediated by an increase in the rate of excitatory and inhibitory synaptic inputs [21], [31], in a fashion similar to the case presented in our study. Dual intracellular experiments that measure both input and output correlation across distinct neural states [45], [46], [37] are required to parcel the contribution of correlation inheritance and correlation transfer to the full shift in noise correlations.

### Connecting Single Neuron and Network Modulations

A central result of our paper is that changes in synaptic input rate shape the correlation between the output spike trains from a pair of neurons. This is a consequence of how synaptic input modulates the timescale of membrane integration and response sensitivity of the two neurons. Our theoretical analysis formalizes this concept by explicitly relating the spike train correlation coefficient to the single neuron transfer function. Though we focused on modulation by balanced synaptic inputs, the relationship between transfer function and correlation is general, requiring only that the input correlation be sufficiently small. Thus, we predict that any synaptic or cellular mechanism that modulates single neuron transfer will necessarily affect spike train correlations.

Modulation of single neuron transfer with the level of synaptic input rate is well studied [27], [28], [29], [30], [31], [32]. However, how other cellular processes affect neuronal transfer is equally well studied. For example, increases in the spike after-hyperpolarization [50] or decreases in the spike after-depolarization [51] reduce the gain of the firing rate response to static driving inputs. Sustained firing often recruits slowly activating adaptation currents that also reduce gain [52], [53]. We predict that these modulations will reduce long timescale spike rate correlations. In contrast, the presence of low threshold potassium currents in the auditory brainstem [71] promotes high frequency single neuron transfer and thus may also promote pairwise synchronization. In total, our result gives a general theory that links the modulation of single neuron and network responses, thereby expanding the applicability of studies of single neuron modulation.

### Selective Propagation of Neural Activity

How the brain selectively propagates signals is a basic question in systems neuroscience. One control mechanism is through an ‘unbalancing’ of feedforward excitation to inhibition, with disinhibited populations propagating activity and excessive inhibition silencing propagation [72]. Modulation of correlation is an alternative mechanism to control signal propagation. The correlation between spike trains from neurons in a population enhances the ability of that population's activity to drive downstream targets [12], [13]. We have shown that modulating the timescale of correlation in the upstream population to match the integration timescale of the downstream population improves signal propagation (Figure 7). Matching the integration dynamics of distinct neuronal populations to one another is a common theme in the binding of distributed activity [73]. In previous studies, the phase relationship between distinct neuronal populations both oscillating at some frequency gated the interaction between distinct brain regions. Our study did not assume rhythmic population dynamics, but rather only matched integration timescales.

The nonlinearity of spike generation allows for the transfer of shared input to multiple neurons to be controlled in complex ways. We have shown that well-studied mechanisms of single neuron response modulation, such as firing rate gain control, have direct relations to changes in correlation for neuron pairs. Thus, state dependent shifts in single neuron transfer also influence how populations of neurons coordinate their activity. Our results are a step in understanding how the collective behavior of neuronal networks can be controlled in different brain states.

## Supporting Information

Figure S1Diffusion limit shows qualitative effects of correlation shaping. (A) Top: Correlation in the low and high states for a conductance-based model with alpha-function synapses. The excitatory time constant was 2.5 ms and the inhibitory time constant 5 ms. The amplitude of the alpha function was taken so that it matched with the delta-function synapses described in the main text. Other parameters were as in the main text. Bottom: Ratio of correlations between the high and low states. (B) Same as (A), but after taking the diffusion approximation (see Eq. 1 in the main text). (C) Same as (B), but after taking 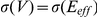. The ratio 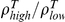 exhibits similar correlation shaping in all cases.(PDF)

Figure S2 Comparison between simulation and experimental results. (A) Top: Correlation in the low and high states calculated from dynamic clamp experiments. Bottom: Ratio of correlations between the high and low states. (B) Similar to (A), showing results from a conductance-based model with alpha-function synapses. The excitatory time constant was 6 ms and the inhibitory time constant 8 ms. The firing rate was 5 Hz to match experiments. Other parameters were as in the main text.
(PDF)

Figure S3Results hold for large 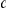. Top: Correlation in low and high states for 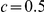, parameters otherwise identical to Figure 3 in the main text. Bottom: Ratio of correlations in the low and high states.(PDF)

Figure S4Change in power spectrum of the spike train 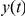 from low to high states. In both cases, the high-frequency limit of the power spectrum is equal to the firing rate of the neuron. For low frequencies, however, the power was increased in the high state, reflecting the increased variability of firing in the high state (note that as frequency
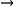
0, the power spectrum is equal to the firing rate multiplied by the square of the inter-spike interval CV). To determine the denominator of Eq. (7), we integrate the power spectrum by 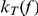 to obtain 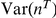. When 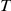 is small, 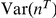 is identical in the two states, because the high frequency limits of the power spectrum are equal. When 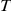 is large, 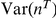 is increased in the high state, because the low frequency limit of the power spectrum is enhanced.(PDF)

Figure S5Correlation shaping occurs for different synaptic strengths. (A) Theoretically calculated correlation curve for 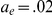, 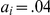, 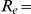
1 kHz in the low state and 4.08 kHz in the high state. The time constant decreased from 7.5 ms in the low state to 1.9 ms in the high state. Firing rates were 15 Hz in both states. (B) Theoretically calculated correlation curve for 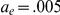, 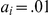, 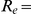
2 kHz in the low state and 8 kHz in the high state. The time constant decreased from 15.2 ms in the low state to 4.8 ms in the high state. Firing rates were 15 Hz in both states.(PDF)
