# Supplementary figures and images for: Balanced Synaptic Input Shapes the Correlation between Neural Spike Trains
Source: PLoS Comput Biol. 2011 Dec 22;7(12):e1002305. doi: 10.1371/journal.pcbi.1002305 (PMC3245294; doi:10.1371/journal.pcbi.1002305)

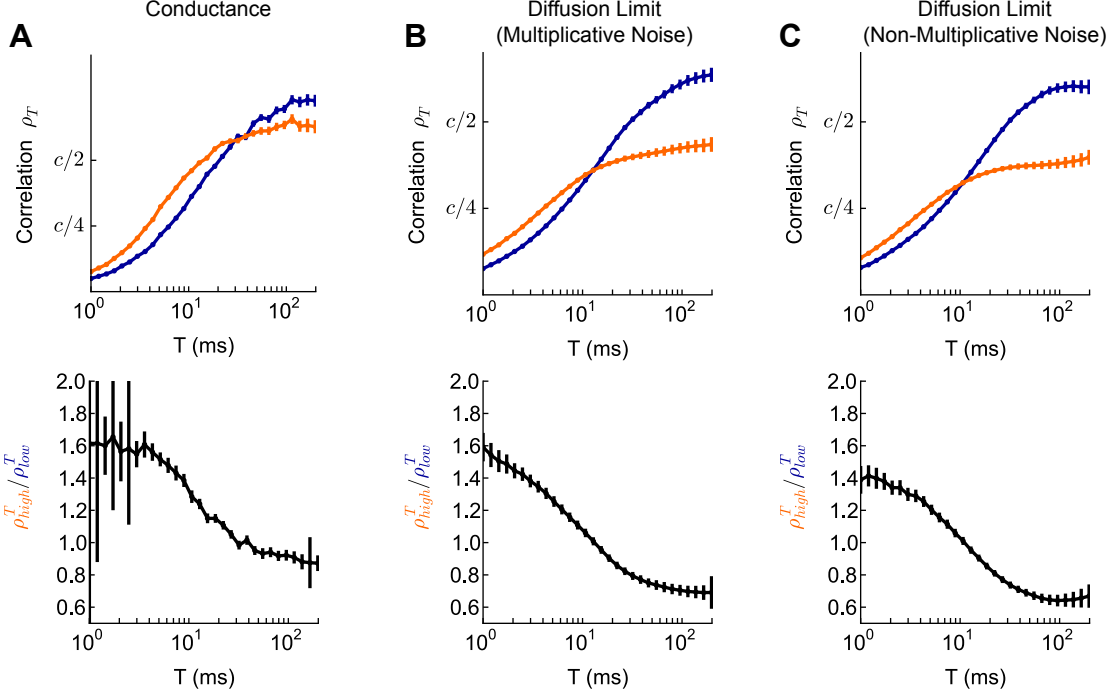

Supplement: Figure S1 — Diffusion limit shows qualitative effects of correlation shaping. (A) Top: Correlation in the low and high states for a conductance-based model with alpha-function synapses. The excitatory time constant was 2.5 ms and the inhibitory time constant 5 ms. The amplitude of the alpha function was taken so that it matched with the delta-function synapses described in the main text. Other parameters were as in the main text. Bottom: Ratio of correlations between the high and low states. (B) Same as (A), but after taking the diffusion approximation (see Eq. 1 in the main text). (C) Same as (B), but after taking . The ratio exhibits similar correlation shaping in all cases. (PDF) [file pcbi.1002305.s001.pdf]

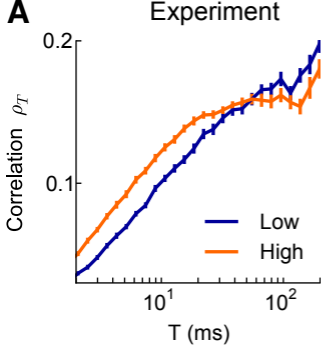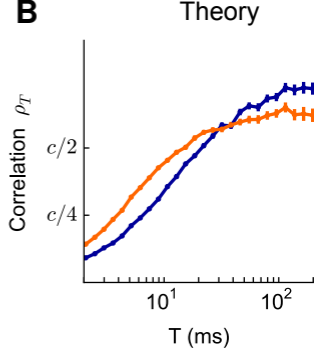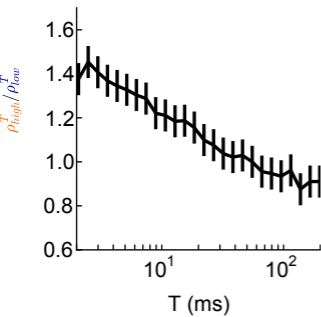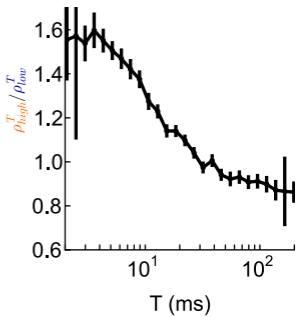

Supplement: Figure S2 — Comparison between simulation and experimental results. (A) Top: Correlation in the low and high states calculated from dynamic clamp experiments. Bottom: Ratio of correlations between the high and low states. (B) Similar to (A), showing results from a conductance-based model with alpha-function synapses. The excitatory time constant was 6 ms and the inhibitory time constant 8 ms. The firing rate was 5 Hz to match experiments. Other parameters were as in the main text. (PDF) [file pcbi.1002305.s002.pdf]

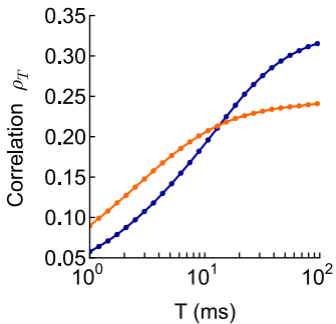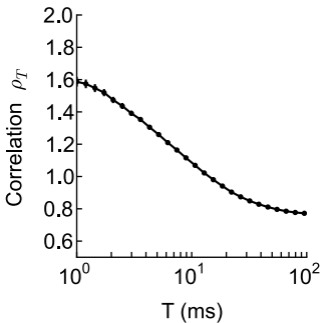

Supplement: Figure S3 — Results hold for large . Top: Correlation in low and high states for , parameters otherwise identical to Figure 3 in the main text. Bottom: Ratio of correlations in the low and high states. (PDF) [file pcbi.1002305.s003.pdf]

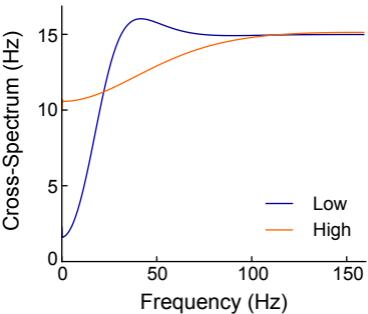

Supplement: Figure S4 — Change in power spectrum of the spike train from low to high states. In both cases, the high-frequency limit of the power spectrum is equal to the firing rate of the neuron. For low frequencies, however, the power was increased in the high state, reflecting the increased variability of firing in the high state (note that as frequency0, the power spectrum is equal to the firing rate multiplied by the square of the inter-spike interval CV). To determine the denominator of Eq. (7), we integrate the power spectrum by to obtain . When is small, is identical in the two states, because the high frequency limits of the power spectrum are equal. When is large, is increased in the high state, because the low frequency limit of the power spectrum is enhanced. (PDF) [file pcbi.1002305.s004.pdf]

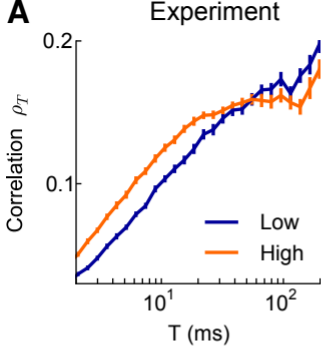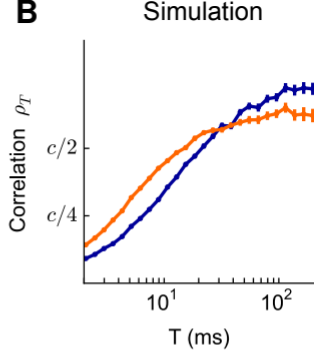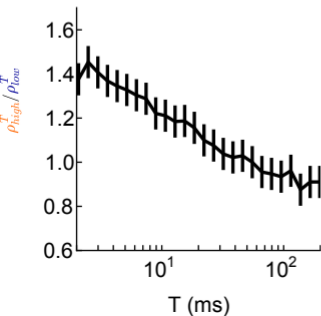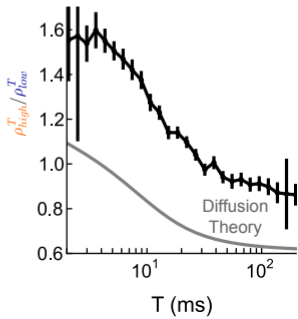

Supplement: Figure S5 — Correlation shaping occurs for different synaptic strengths. (A) Theoretically calculated correlation curve for , , 1 kHz in the low state and 4.08 kHz in the high state. The time constant decreased from 7.5 ms in the low state to 1.9 ms in the high state. Firing rates were 15 Hz in both states. (B) Theoretically calculated correlation curve for , , 2 kHz in the low state and 8 kHz in the high state. The time constant decreased from 15.2 ms in the low state to 4.8 ms in the high state. Firing rates were 15 Hz in both states. (PDF) [file pcbi.1002305.s005.pdf]
